# SARS-CoV-2 in Pregnant Women: Consequences of Vertical Transmission

**DOI:** 10.3389/fcimb.2021.717104

**Published:** 2021-09-09

**Authors:** Ishaan Chaubey, Ramachandran Vignesh, Hemalatha Babu, Isabelle Wagoner, Sakthivel Govindaraj, Vijayakumar Velu

**Affiliations:** ^1^The Center for Advanced Studies in Science, Math, and Technology at Wheeler High School, Marietta, GA, United States; ^2^Preclinical Department, Faculty of Medicine, Royal College of Medicine Perak, Universiti Kuala Lumpur, Ipoh, Malaysia; ^3^Department of Pathology and Laboratory Medicine, Emory University School of Medicine, Atlanta, GA, United States; ^4^Division of Microbiology and Immunology, Yerkes National Primate Research Center, Emory Vaccine Center, Emory University, Atlanta, GA, United States

**Keywords:** SARS-CoV-2, pregnant women, vertical transmission, COVID-19, mother to child

## Introduction

Beginning in December 2019, multiple cases of pneumonia were reported in the city of Wuhan, China, but further scans of the respiratory tract and the genetic sequencing of infected individuals revealed the presence of a novel coronavirus, later designated as SARS-CoV-2 (Gao et al., 2020); the subsequent disease, now widely known as COVID-19. As the virus rapidly spread across the world, the World Health Organization (WHO) declared a global pandemic emergency, but many researchers saw a silver lining since it was found that the SARS-CoV-2 had a lower mortality rate as compared to SARS-CoV (the cause of the SARS outbreak in 2003) and MERS-CoV (Ellington et al., 2020; Fani et al., 2020). As of August 5^th^ 2021, there were a total of 201,268,189 COVID-19 cases with 4,275,004 deaths across the world.

Studies have shown that there are certain groups of people who are at an increased risk for acquiring this viral infection, and one of these groups is pregnant women. Though pregnant women are less likely to experience the headache, muscle aches, fever, chills, and diarrheal symptoms of COVID-19, they are more prone to failing respiratory or cardiovascular systems, especially if they have underlying conditions such as chronic lung disease or cardiovascular disease (Ellington et al., 2020). Additionally, existing data illustrate that the age of an infected woman plays a role in the severity of COVID-19 symptoms (Ellington et al., 2020). For instance, among infected women of the reproductive age range (ages 12-51), it was found that pregnant women in this range were more likely to be hospitalized and admitted into the Intensive Care Unit (ICU) as compared to non-pregnant women in the same range, but both non-pregnant and pregnant women had a similar chance for death (Ellington et al., 2020; Zambrano et al., 2020). Since pregnant women are considered to be vulnerable to SARS-CoV-2 infection, it is essential to understand if the virus could infect the developing fetus (Panagiotakopoulos et al., 2020; Brandt and Fell, 2021; Vivanti et al., 2020a; Vouga et al., 2021).

The SARS-CoV-2 virus specifically utilizes the angiotensin-converting enzyme-2 (ACE2) cell receptor and the serine protease TMPRSS2 enzyme for cell entry (Pique-Regi et al., 2020). In the case of pregnant women, the single-cell transcriptomic analysis performed in the study by Pique-Regi et al. (2020) showed that ACE2 and TMPRSS2 were minimally expressed in the placenta throughout pregnancy, and hence, it was inferred that the chances of placental infection were unlikely. However, it is to be noted that several other studies demonstrated the possibility of placental infection by SARS-CoV-2 (Baud et al., 2020; Fenizia et al., 2020; Hosier et al., 2020; Pulinx et al., 2020; Vivanti et al., 2020b; Zhu et al., 2020; Zhou et al., 2021).

A case study by Hosier et al. (2020) found high levels of SARS-CoV-2 viral RNA in the placenta and umbilical cord of a SARS-CoV-2 infected pregnant woman, indicating evidence of SARS-CoV-2 invasion in the maternal and fetal tissues (Hosier et al., 2020). However, no definitive evidence of fetal infection from SARS-CoV-2 was found. In another case study by Pulinx et al., 2020 (Pulinx et al., 2020), involving a SARS-CoV-2 infected pregnant woman with mild COVID-19 symptoms, placental tissue samples, amniotic fluid, and the maternal blood samples at the time of birth tested positive for SARS-CoV-2 (Pulinx et al., 2020). From these two case studies, it was seen that SARS-CoV-2 does indeed infect the placenta of pregnant women, but there was no conclusive evidence that SARS-CoV-2 is vertically transmitted resulting in fetal infection. However, it should be noted that both Hosier et al. (2020) and Pulinx et al. (2020) reported evidence of SARS-CoV-2 viral infection in the maternal tissues, amniotic fluid, and maternal blood samples during the pregnancy process. Therefore, it remains vital to understand the viral pathogenesis in specific anatomical locations in the bodies of infected pregnant women and hence determine if SARS-CoV-2 can vertically transmit from an infected mother to her child. Hence, in this review, we aimed to analyze the virological and immunological perspectives of SARS-CoV-2 infection in infected pregnant women and discuss the consequences of COVID-19 in the mother and infants.

## Dynamics of Vertical Transmission Between Mother and Child

In addition to documenting the presence of SARS-CoV-2 in the placenta, the case studies by Hosier et al. (2020) and Pulinx et al. (2020) have specifically shown that the virus infects the Syncytiotrophoblast cells at the highest rate. Since the Syncytiotrophoblast cells of the placenta are responsible for the circulation of nutrients between the mother and fetus (Aplin, 2010; Arora et al., 2017; Komine-Aizawa et al., 2020), higher viral densities in these cells raise the concerns and the possibility of facilitating transmission of the virus to the fetus. However, there are several conflicting reports as to whether a child becomes infected with the SARS-CoV-2 virus as a consequence of vertical transmission. In a case study conducted in Wuhan, China, researchers observed the presence of SARS-CoV-2 in 30 neonates who were born to 29 pregnant women with COVID-19 (Wu Y.T. et al., 2020). Amongst these 30 newborns, 18 were suspected of harboring SARS-CoV-2 and were immediately placed under quarantine and care while the remaining 12 were normally discharged after birth with a regular follow-up. Of the 18 newborns suspected of having the virus, 5 newborns were diagnosed with COVID-19, and 12 others possessed no COVID-19 symptoms but presented several radiological features of pneumonia through X-Ray and CT screening, hence illustrating the possibility of vertical transmission in newborns (Wu Y.T. et al., 2020). While the last remaining newborn had an occasional cough, the researchers were unsure of its SARS-CoV-2 infection status. (Wu Y.T. et al., 2020). In another study conducted by Facchetti et al. (2020), more evidence of SARS-CoV-2 vertical transmission was found when an infected full-term pregnant woman gave birth to a newborn that eventually tested positive for SARS-CoV-2 and developed symptoms for pneumonia. From this study, researchers detected SARS-CoV-2 viral RNA in several maternal-fetal interface cells including the Syncytiotrophoblast cells and identified multiple SARS-CoV-2 virus particles infecting fetal intravascular monocytes. Subsequently, it was inferred that the infected circulating fetal blood cells may have served as the vehicles for the SARS-CoV-2 virus to travel from the infected mother to the fetus and consequently lead to infection in the newborn. In addition, a separate study by (Pique-Regi et al., 2020) showed that a small fraction of neonates born to women with COVID-19 tested positive for the virus at 1–4 days of life, but these neonates subsequently tested negative on days 6-7, potentially indicating that had vertical transmission occurred, the virus remained short-lived within the bodies of these newborns. Hence, it becomes essential to understand the immune responses against SARS-CoV-2 in both pregnant women and their developing fetuses.

## Antibody Response Against SARS-CoV-2

In the humoral arm of adaptive immune responses against SARS-CoV-2 infection, three different classes of immunoglobulins namely, IgM, IgG, and IgA are involved. IgM antibodies are the first set of antibodies generated in response to viral infection, and they are the largest antibodies that are commonly found in blood and lymph fluid (Ma et al., 2020). IgA antibodies are found in areas of the body which are frequently exposed to foreign pathogens such as the nasal cavities, breathing passages, digestive tract, ears, eyes, and vagina as well as certain bodily fluids such as saliva, tears, and blood (CDC COVID-19 Science Briefs, 2020). IgA antibodies also play a crucial role against SARS-CoV-2 viral infection since these antibodies are generated shortly after IgM antibodies are secreted, and IgA antibodies serve as the first line of immune defense by preventing SARS-CoV-2 viral particles from infecting several critical regions of the human body such as the respiratory tract, digestive tract, vagina, and so forth (Ma et al., 2020). (CDC COVID-19 Science Briefs, 2020; Chao et al., 2020). Lastly, IgG antibodies are the smallest and most prevalent antibodies found in all body fluids, and these antibodies are generated a few days after IgA antibodies are released to combat the SARS-CoV-2 virus (Ma et al., 2020). As of May 2021, little is truly known about how long IgM, IgG, and IgA antibodies persist in the human body to combat SARS-CoV-2 infection. However, it was shown in a study conducted by (Iyer et al., 2020) that IgM and IgA antibodies declined after initial SARS-CoV-2 infection, but anti-SARS-CoV-2 IgG antibodies persisted for at least 90 days after initial onset of COVID-19 symptoms. In addition, it was found that the persistence of SARS-CoV-2 specific IgG antibodies provided sufficient protection against future SARS-CoV-2 infections (Chandrashekar et al., 2020), hence signifying the idea that infected patients were less vulnerable to future SARS-CoV-2 infections. Nonetheless, in these rare cases of SARS-CoV-2 reinfection, it was found that very few cases of reinfection resulted in a severe outcome, and the majority of SARS-CoV-2 reinfection cases were mild or moderate (Cohen and Burbelo, 2020; Selvaraj et al., 2020; Abu-Raddad et al., 2021). Nonetheless, it was found in a study conducted by (Hu et al., 2020) that the severity of the COVID-19 disease influenced the increase in the levels of IgM, IgA and IgG antibodies.

### COVID-19 Disease Severity and Its Effects on IgM and IgG Antibody Production

The severity of COVID-19 is classified into three categories: Mild, Moderate, and Severe, and each classified category consists of a few specific symptoms. Moreover, patients with moderate to severe symptoms of COVID-19 are hospitalized to prevent further progression of the disease. Interestingly, a study showed that IgM antibody levels for patients with moderate to severe COVID-19 were higher for the first 14 days after the onset of COVID-19 symptoms as compared to the levels for those with mild COVID-19 (Hu et al., 2020). However, after the first 14-day period, the IgM levels of mild patients surpassed the IgM levels of moderate and severe patients (Hu et al., 2020). When IgG antibody levels were compared between patients with mild symptoms and patients with moderate to severe symptoms, it was found that moderate and severe patients consistently had higher IgG levels than mild patients (Hu et al., 2020; Joseph et al., 2021). Therefore, this study highlighted the association between the severity of COVID-19 symptoms and the levels of IgM and IgG antibodies in infected patients.

Regarding SARS-CoV-2 infected pregnant women, disease severity could have influenced the vertical transmission of the virus. In the study by Hu et al. (2020) (Hu et al., 2020), different levels of IgM and IgG antibodies were released to combat the SARS-CoV-2 viral infection depending on disease severity. During pregnancy, IgG antibodies are the only class of antibodies that can transfer from the placenta to the fetus owing to their small molecular size whereas IgM antibodies are unable to cross the placenta due to their larger molecular weight. However, two recent case studies indicated that a few newborns who were successfully delivered from their infected mothers had increased amounts of IgM and IgG antibodies (Zeng et al., 2020; Dong et al., 2020; Zeng et al., 2020). Since IgM antibodies are the first component of the immune response against SARS-CoV-2, the presence of IgM antibodies in these newborns indicates the possibility of them being recently exposed and infected with the SARS-CoV-2 virus as fetuses in the womb (Pique-Regi et al., 2020). However, the very presence of IgM antibodies does not concretely imply that the fetuses were infected and is not evident of vertical transmission. In addition, Gao et al. (2020) found that the occurrence of neonatal infection or neonatal asphyxia in neonates born to SARS-CoV-2 infected mothers was not statistically significant, indicating that transmission of the SARS-CoV-2 virus from the placenta to a fetus is a rare occurrence during pregnancy. Therefore, to effectively identify the potential consequences of SARS-CoV-2 vertical transmission, it is prudent to determine the virological and immunological characteristics of SARS-CoV-2 infection in pregnant women. Since a few studies have already illustrated that infected fetuses or newborns possess IgM antibodies for combating viral infection (Pulinx et al., 2020; Wu Y.T. et al., 2020), it is important to understand the conditions in pregnant women which enable SARS-CoV-2 to vertically transmit from an infected mother to her child. Additionally, if vertical transmission did indeed occur from an infected mother to her fetus, this study also analyzed the implications for the growth and development of the fetus or newborn over a period of time.

## Pathology of SARS-CoV-2 Infection in Pregnant Women

Pregnant women have been reported to generally experience mild to moderate COVID-19 symptoms with few cases progressing to severe COVID-19 (Breslin et al., 2020). However, it is to be noted that pregnant women with SARS-CoV-2 infection were more susceptible to several pregnancy complications such as preeclampsia during the delivery process. In addition, a few studies illustrated that the SARS-CoV-2 virus almost consistently targeted the placenta for its replication, more specifically targeting the Syncytiotrophoblast cells for its replication (Baud et al., 2020; Fenizia et al., 2020; Hosier et al., 2020; Pulinx et al., 2020; Vivanti et al., 2020b). Furthermore, the ACE2 receptor, which is critical for SARS-CoV-2 entry into the host cells, was frequently expressed in the maternal-fetal interface cells, hence justifying why the Syncytiotrophoblast cells in the placenta were primarily targeted by SARS-CoV-2 for viral replication (Fenizia et al., 2020; Golden and Simmons, 2020; Lai et al., 2020). Additionally, it was inferred that the presence of SARS-CoV-2 in these critical Syncytiotrophoblast cells may have led to intervillosis (miscarriage) in infected pregnant women (Baud et al., 2020; Fenizia et al., 2020; Hosier et al., 2020; Pulinx et al., 2020; Vivanti et al., 2020b) and occasional death in newborns as observed in several studies (Knight et al., 2020; Pulinx et al., 2020; Zhu et al., 2020)) since these cells played a significant role in the exchange of vital nutrients, antibodies, and Oxygen between the mother and child (Aplin, 2010). In addition, a single-cell transcriptome study revealed high expression of ACE2 in maternal-fetal interface cells in placenta and also in specific cell types of fetal heart, liver and lung. (Li et al., 2020). A recent study analyzing placentas from SARS-CoV-2 infected deliveries reported detection of the SARS-CoV-2 spike protein in all the cases and in all of the compartments at the maternal-fetal interface, including trophoblasts, decidual stromal cells, immune cells, epithelial cells of the placenta and amnion and endothelial cells of the umbilical cord (Verma et al., 2021). Interestingly, a recent study suggests the potential of ACE-2 expressing immune cells in maternal circulation to traffic SARS-CoV-2 to the placenta and possibly increase the risk of vertical transmission. (Lye P. et al., 2021).

Furthermore, since ACE2 expression changes significantly in the maternal-fetal interface cells in the different trimesters of pregnancy (Fenizia et al., 2020), the majority of case studies and literature reviews indicated that many pregnant women who had possibly transmitted the SARS-CoV-2 virus to their fetus were in their third trimester (Bellos et al., 2021). However, a study conducted by Edlow et al. (2020) found that it was unlikely for pregnant women in their third trimester to transmit SARS-CoV-2 to their developing newborn. It has also been shown that severe SARS-CoV-2 placenta infection can impact neonatal outcome in the absence of vertical transmission (Cribiu et al., 2021). Therefore, further research is necessary to effectively determine if the different trimesters of pregnancy could influence the vertical transmission of the SARS-CoV-2. Additionally, a few studies have reported that besides infecting the placenta, the SARS-CoV-2 virus also targeted the umbilical cord for its replication, consequently resulting in the occasional inflammation of the umbilical cord as well as the presence of several viral densities of SARS-CoV-2 (Baud et al., 2020; Fenizia et al., 2020; Hosier et al., 2020).

Another recent case study of a still-born fetus of a pregnant woman with COVID-19 revealed the placenta and umbilical cord blood testing positive for SARS-CoV-2 RNA, and pathological findings of fetal tissues were suggestive of vascular damage caused by infection (Stonoga et al., 2021). In addition, findings have illustrated that a swab of the vagina or a swab of the nasopharynx possessed large amounts of SARS-CoV-2 viral RNA (Baud et al., 2020; Fenizia et al., 2020; Vivanti et al., 2020b). Eventually, through RT-PCR testing after the human birthing process, the maternal blood and the amniotic fluid have also shown to possess SARS-CoV-2 viral RNA (Pulinx et al., 2020; Vivanti et al., 2020b; Zamaniyan et al., 2020). Therefore, these works of literature clearly reveal that there were several other regions in the body of an infected pregnant woman that could be capable of vertically transmitting SARS-CoV-2 during the time of birth and subsequently result in fetal infection (Baud et al., 2020; Fenizia et al., 2020; Hosier et al., 2020; Pulinx et al., 2020; Vivanti et al., 2020b; Zamaniyan et al., 2020).

## Immunopathogenesis of SARS-CoV-2 Infection in Pregnant Women

Regarding the immunology of SARS-CoV-2 infection in pregnant women, studies have demonstrated elevated levels of anti-SARS-CoV-2 IgM and IgG antibodies (Dong et al., 2020; Iyer et al., 2020), and it was inferred that the increased amount of IgM and IgG antibodies was due to the immune response of infected pregnant women actively combating SARS-CoV-2. It is also of importance to ponder the possible pathogenic role of antibodies in facilitating the entry of virus through antibody dependent enhancement (ADE) as observed in SARS and MERS viruses (Moore and Suthar, 2021). Studies have also indicated the prevalence of an exaggerated immune response known as the cytokine storm (Dhama et al., 2020; Fenizia et al., 2020; Liu H. et al., 2020; Narang et al., 2020), a sudden release of a surge of inflammatory cytokine molecules which eventually results in the human body attacking its own cells and tissues and thereby leading to several adverse health complications (Soy et al., 2020; Ye et al., 2020). In the case of SARS-CoV-2 infection, it was found that the sudden surge of inflammatory cytokines in a cytokine storm led the human immune system to attack several critical pulmonary tissues as well as other significant cellular tissues, rather than the SARS-CoV-2 virus itself, resulting in the occasional increase in COVID-19 disease severity from mild or moderate to severe disease in infected pregnant women and intermittent death depending on the extent of cellular and tissue damage (Dhama et al., 2020; Fenizia et al., 2020; Liu H. et al., 2020; Narang et al., 2020). Furthermore, several reports have shown elevated levels of C-reactive protein (CRP) in infected pregnant women (Areia and Mota-Pinto, 2020; Chen et al., 2020; Wu C. et al., 2020), and it was inferred that high levels of CRP led to premature birth in newborns born to SARS-CoV-2 -infected mothers (Xiong et al., 2020). Furthermore, earlier literature has demonstrated the role of increased CRP levels in preterm labor and premature rupture of membranes in pregnant women (Najat Nakishbandy and Barawi, 2014). CRP is a nonspecific biomarker of inflammation which contributes for preterm birth (Huang et al., 2020). Furthermore, significantly varied levels of inflammatory biomarker CRP was observed in SARS-CoV-2 affected pregnant as well as postpartum women, inflammation caused by SARS-CoV-2 which may contribute for preterm birth (Khalil et al., 2020; Woodworth et al., 2020). Along with the elevated levels of CRP, increased amounts of D-dimer have also been observed in infected pregnant women (Areia and Mota-Pinto, 2020; Zhou et al., 2021), and research suggested that elevated levels of D-dimer may have led to the progression of COVID-19 disease severity in several pregnant women since high amounts of D-dimer signified an increased chance of acquiring Acute Respiratory Distress Syndrome (ARDS) and being admitted into the ICU and dying (Vidali et al., 2020). A recent study has demonstrated the significant impact of COVID-19 infection on cytokine profile of pregnant women and that the cytokine levels being correlating with the severity of disease (Tanacan et al., 2020). These characteristics indicate the intense inflammatory processes mediating the pathogenesis of COVID-19 that follows the disease severity. Likewise, a recent study has shown significant depletion of selenium among pregnant women with COVID-19 and that it correlates with the levels of IL-6 and D-dimer thereby suggesting its possible role in disease progression. In addition, there are multiple pieces of evidence suggesting that pregnant women infected with SARS-CoV-2 frequently possessed lymphopenia or a lower number of B lymphocytes, T lymphocytes, and neutralizing natural killer cells as compared to their non-infected counterparts (Chen et al., 2020; Narang et al., 2020; Wu C. et al., 2020) which was greatly concerning because these white blood cells play a critical role in the immune response against several pathogens including SARS-CoV-2 (Liu J. et al., 2020; Sheu and Chiang, 2021).

## Implications of SARS-CoV-2 Vertical Transmission on Developing Fetus or Newborn

Finally, in regards to the implications of SARS-CoV-2 vertical transmission on fetuses or newborns, several studies found that many newborns presented mild to moderate COVID-19 symptoms such as fever, shortness of breath, a pulmonary infection like pneumonia, and various gastrointestinal symptoms. In contrast, a few studies found that newborns tended to be asymptomatic after delivery (Golden and Simmons, 2020; Knight et al., 2020; Liu H. et al., 2020; Vivanti et al., 2020b; Wu Y.T. et al., 2020; Zhu et al., 2020; Zhou et al., 2021). Rarely, it was found that mild to moderate COVID-19 symptoms progressed to severe COVID-19 in newborns, and within these few cases, newborns were found to have neonatal respiratory distress syndrome (NRDS) and eventually perish (Knight et al., 2020; Pulinx et al., 2020; Zhu et al., 2020). Hence, in cases suggestive of SARS-CoV-2 vertical transmission, the majority of infected newborns had a chance for a healthy recovery and were not as adversely impacted by viral infection. However, in the few instances in which newborns infected with SARS-CoV-2 progressed to severe COVID-19 symptoms, it was inferred that certain pre-existing conditions present within the newborn might have influenced COVID-19 severity, but more investigation into this topic is necessary to derive an accurate conclusion.

Also, it was found that newborns infected with SARS-CoV-2 tended to possess higher levels of anti-SARS-CoV-2 IgM and IgG antibodies illustrating that their immune system was actively combating SARS-CoV-2 (Dong et al., 2020; Golden and Simmons, 2020; Vivanti et al., 2020b; Zhou et al., 2021). Furthermore, the presence of IgM antibodies in these newborns indicated that these neonates were recently infected *in utero* or during the birthing process since IgM antibodies are unable to cross the placenta (Pique-Regi et al., 2020). Hence, this common finding justified that viral infection could occur in several other anatomical regions besides the placenta since the newborn could be infected with SARS-CoV-2 through the umbilical cord, nasopharynx, maternal blood, amniotic fluid, and the vagina, which are all key regions involved during the birthing process (Baud et al., 2020; Fenizia et al., 2020; Hosier et al., 2020; Pulinx et al., 2020; Vivanti et al., 2020b; Zamaniyan et al., 2020). This study also found that there was an increased proportion of leukocytes, neutrophils, and lymphocytes in the newborn against SARS-CoV-2 indicating increased immune response activity against the virus (Dong et al., 2020; Golden and Simmons, 2020; Vivanti et al., 2020b; Zhou et al., 2021). However, it should be noted that in the small number of cases in which newborns progressed to severe SARS-CoV-2 infection, it was highly likely that these newborns were immunocompromised through an underlying condition, but since several existing pieces of literature did not account for this detail, further research is necessary to determine if specific underlying conditions amongst newborns could truly lead to an increased chance for severe COVID-19 and eventual death. A recent study by us revealed that there is a significant drop in the level of RBD specific binding and neutralizing antibodies in cord blood compared to the maternal blood. These lower responses in cord may pose a threat to newborns (Joseph et al., 2021). In addition, for some newborns with SARS-CoV-2 infection, studies have indicated that SARS-CoV-2 influenced the development of the neonatal nervous system and led to various neuronal dysfunctions, and neonatal encephalopathy (NE) along with several other mental health complications (Knight et al., 2020; Liu H. et al., 2020). Hence, it was inferred that SARS-CoV-2 made the fetus or newborn more susceptible to future mental illnesses and neurological disorders, but more research is needed to derive a conclusion in regards to the long-term implications of SARS-CoV-2 infection on the neonatal brain and nervous system development. Moreover, there are findings of newborns having abnormalities in their livers after being infected with SARS-CoV-2 as a result of possible vertical transmission (Dong et al., 2020; Zhu et al., 2020) possibly hinting at the consequence of various gastrointestinal symptoms of COVID-19 experienced by the newborns [**Table 1**; (Dong et al., 2020; Golden and Simmons, 2020; Liu H. et al., 2020; Zhu et al., 2020)]. In addition, another recent study has indicated that SARS-CoV-2 in the third trimester of pregnancy could cause fetal kidney developmental injury (He et al., 2021). Finally, studies have also illustrated that SARS-CoV-2 infected newborns tended to have faster heart rates or tachycardia or various heart abnormalities (Golden and Simmons, 2020; Liu H. et al., 2020; Zhu et al., 2020). This implies the possibility of SARS-CoV-2 infection leading to future heart complications, but further research into this topic is necessary as well.

**Table 1:** Consequences of SARS-CoV-2 Infection and Transmission in pregnant women.

SARS-CoV-2 infection in nasopharynx	Baud et al., 2020; Dong et al., 2020; Pulinx et al., 2020; Vivanti et al., 2020b; Saccone et al., 2021
SARS-CoV-2 infection in lungs; Patchy or hazy opacities near or on lungs	Dong et al., 2020; Wu C. et al., 2020; Xiong et al., 2020; Zhou et al., 2020; Zhu et al., 2020; Cruz-Lemini et al., 2021
SARS-CoV-2 infection in placenta; Intervillosis observed in placenta; Abnormalities in the placenta	Baud et al., 2020; Fenizia et al., 2020; Hosier et al., 2020; Naz et al., 2020; Pulinx et al., 2020; Vivanti et al., 2020b; Zhou et al., 2020; Zhu et al., 2020
SARS-CoV-2 infection in umbilical cord; Abnormalities and/or inflammation of umbilical cord	Baud et al., 2020; Fenizia et al., 2020; Hosier et al., 2020; Naz et al., 2020; Zhu et al., 2020
SARS-CoV-2 infection in maternal blood	Fenizia et al., 2020; Pulinx et al., 2020; Vivanti et al., 2020b
SARS-CoV-2 infection in amniotic fluid	Fenizia et al., 2020; Pulinx et al., 2020; Vivanti et al., 2020b; Zamaniyan et al., 2020; Zhu et al., 2020
Mild to moderate (occasionally severe) COVID-19 disease symptoms in infected pregnant women	Baud et al., 2020; Breslin et al., 2020; Chen et al., 2020; Chi et al., 2020; Dong et al., 2020; Hosier et al., 2020; Knight et al., 2020; Lai et al., 2020; Liu H. et al., 2020; Narang et al., 2020; Pulinx et al., 2020; Wu C. et al., 2020; Wu Y.T. et al., 2020; Xiong et al., 2020; Zamaniyan et al., 2020; Zhou et al., 2020; Zhu et al., 2020; Bellos et al., 2021; Di Toro et al., 2021; Saccone et al., 2021
Cytokine storm involved in immune response in infected pregnant women against SARS-CoV-2; Increased cytokine levels	Dhama et al., 2020; Fenizia et al., 2020; Liu H. et al., 2020; Narang et al., 2020
Lymphopenia observed in infected pregnant women	Areia and Mota-Pinto, 2020; Chen et al., 2020; Chi et al., 2020; Dhama et al., 2020; Hosier et al., 2020; Narang et al., 2020; Vivanti et al., 2020b; Wu C. et al., 2020; Wu Y.T. et al., 2020; Zamaniyan et al., 2020; Bellos et al., 2021; Cruz-Lemini et al., 2021; Di Toro et al., 2021; Saccone et al., 2021
Elevated C-reactive protein levels in SARS-CoV-2 infected pregnant women	Areia and Mota-Pinto, 2020; Chen et al., 2020; Chi et al., 2020; Dhama et al., 2020; Pulinx et al., 2020; Vivanti et al., 2020b; Wu C. et al., 2020; Wu Y.T. et al., 2020; Zamaniyan et al., 2020; Bellos et al., 2021; Di Toro et al., 2021
Increased levels of anti-SARS-CoV-2 IgM and IgG antibodies in pregnant women	Dong et al., 2020; Hosier et al., 2020; Iyer et al., 2020; Zhou et al., 2020
Elevated levels of D-dimer in SARS-CoV-2 infected pregnant women	Areia and Mota-Pinto, 2020; Chi et al., 2020; Dhama et al., 2020; Wu C. et al., 2020; Zhou et al., 2020; Bellos et al., 2021
ACE2 expressed in maternal-fetal interface cells	Chi et al., 2020; Fenizia et al., 2020; Golden and Simmons, 2020; Lai et al., 2020
Preeclampsia and other pregnancy complications	Areia and Mota-Pinto, 2020; Chen et al., 2020; Chi et al., 2020; Golden and Simmons, 2020; Hosier et al., 2020; Liu H. et al., 2020; Narang et al., 2020; Wu Y.T. et al., 2020; Zhou et al., 2020; Bellos et al., 2021; Cruz-Lemini et al., 2021; Saccone et al., 2021
Abnormalities and Complications in Newborns from SARS-CoV-2 Viral Transmission	
Radiological features of pneumonia, pulmonary infection in the lungs, shortness of breath or oxygen deprivation, or respiratory distress present in newborn	Areia and Mota-Pinto, 2020; Golden and Simmons, 2020; Liu H. et al., 2020; Narang et al., 2020; Naz et al., 2020; Vivanti et al., 2020b; Wu Y.T. et al., 2020; Zhu et al., 2020; Chi et al., 2020; Bellos et al., 2021; Cruz-Lemini et al., 2021; Di Toro et al., 2021
Asymptomatic newborns from infected pregnant women	Dong et al., 2020; Fenizia et al., 2020; Naz et al., 2020; Zamaniyan et al., 2020; Bellos et al., 2021; Di Toro et al., 2021; Saccone et al., 2021
Newborn experiencing fever, gastrointestinal, and other mild to moderate symptoms of COVID- 19	Golden and Simmons, 2020; Liu H. et al., 2020; Narang et al., 2020; Naz et al., 2020; Zhu et al., 2020; Bellos et al., 2021; Di Toro et al., 2021
Elevated anti-SARS-CoV-2 IgM and IgG antibody levels in newborn; Increased immune system activation	Chi et al., 2020; Dong et al., 2020; Golden and Simmons, 2020; Naz et al., 2020; Vivanti et al., 2020b; Zhou et al., 2020; Saccone et al., 2021
Abnormalities in liver function for newborn	Dong et al., 2020; Liu H. et al., 2020; Naz et al., 2020; Zhu et al., 2020; Cruz-Lemini et al., 2021
Abnormalities in development of neonatal nervous system	Knight et al., 2020; Liu H. et al., 2020; Vivanti et al., 2020b; Wu Y.T. et al., 2020
Fetal kidney developmental in	He et al., 2021

## Conclusion

With additional analyses conducted within the scope of this study, several studies have illustrated that the SARS-CoV-2 virus primarily infects the Syncytiotrophoblast cells and the other maternal-fetal interface cells because ACE2 is significantly expressed in these maternal-fetal interface cells. Furthermore, reports indicating the involvement of the umbilical cord, nasopharynx, vaginal mucosa, maternal blood, and amniotic fluid apart from the placenta, point at the possibility of vertical transmission of SARS-CoV-2 during the time of birth. Additionally, after analyzing the literature on the immunology of SARS-CoV-2 infection, pregnant women had an altered immune response making them susceptible to severe or critical COVID-19 since they possessed lymphopenia, elevated levels of C-reactive protein and D-dimer, and were prone to cytokine storm. Also, the fetuses or newborns with SARS-CoV-2 infection were observed with several abnormalities in the liver, oddities in the development of the nervous system, and various complications in the heart and kidneys despite mostly possessing mild to moderate symptoms of COVID-19, as a result of possible vertical transmission. Understanding the immunopathology of SARS-CoV-2 infection in pregnant women as well as the implications of SARS-CoV-2 vertical transmission is paramount to focus future researches in devising strategies to prevent transmission of SARS-CoV-2 from an infected mother to child. In addition, this study also throws light on various abnormalities observed in fetuses or newborns due to SARS-CoV-2 infection and the need to determine measures to combat these developmental challenges. However, further detailed and structured studies are required to ascertain if certain factors such as age and presence of underlying conditions of pregnant women could influence the virological and immunological characteristics of SARS-CoV-2 infection in pregnant women. At the same time, pregnant women from certain ethnic communities are more vulnerable to particular underlying conditions due to various social disparities in healthcare, and subsequently, these groups’ increased susceptibility to severe cases of SARS-CoV-2 infection should be taken into account for future studies. Furthermore, future research should investigate the possible role of variants of SARS-CoV-2 in vertical transmission from an infected mother to her fetus since this virus has evolved and mutated a significant number of times since its emergence in December 2019 (van Dorp et al., 2020). Finally, with the release of new vaccines from Pfizer, Moderna, Johnson & Johnson, and Oxford/AstraZeneca, pregnant women should be vaccinated as soon as possible due to their increased susceptibility to severe COVID-19 and take all necessary precautions to prevent SARS-CoV-2 infection to eventually reduce the chances of vertical transmission and avoid any abnormalities in the growth and development of their child.

## Author Contributions

IC, RV, and VV led the writing of this opinion article. HB, IW, and SG assisted in editing the article. All authors contributed to the article and approved the submitted version.

## Funding

VV was supported by CFAR R03 (to VV), Emory University CFAR grant P30 AI050409 and NCRR/NIH base grants P30 RR00165, P51OD011132 (to Y.N.P.R.C.).

## Author Disclaimer

The views, opinions, assumptions, or any other information set out in this article are solely those of the authors and should not be attributed to anyone. The authors salute all the health care workers who are at the front lines of the COVID-19 pandemic and the scientists striving to combat it.

## Conflict of Interest

The authors declare that the research was conducted in the absence of any commercial or financial relationships that could be construed as a potential conflict of interest.

## Publisher’s Note

All claims expressed in this article are solely those of the authors and do not necessarily represent those of their affiliated organizations, or those of the publisher, the editors and the reviewers. Any product that may be evaluated in this article, or claim that may be made by its manufacturer, is not guaranteed or endorsed by the publisher.
